# Supplementary material for: Integrated analysis of the aqueous humor microbiome and lens capsule transcriptome in high myopia cataract: a pilot study
Source: Front Med (Lausanne). 2026 Jun 16;13:1845205. doi: 10.3389/fmed.2026.1845205 (PMC13314463; doi:10.3389/fmed.2026.1845205)
Supplement: Supplementary file 3 [file Data_Sheet_1.zip › 7.Average/1.Community_Structure/heatmap/C372089/Phylum_top30_nocluster.pdf]

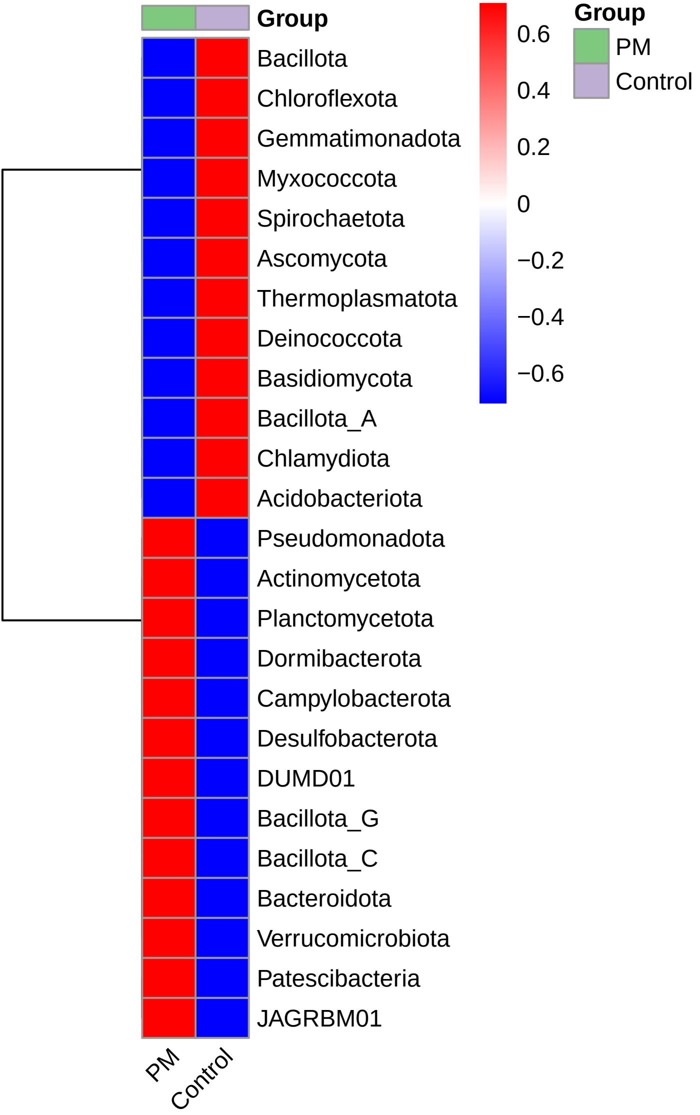
Group
Bacillota
Chloroflexota
Gemmatimonadota
Myxococcota
Spirochaetota
Ascomycota
Thermoplasmatota
Deinococcota
Basidiomycota
Bacillota_A
Chlamydiota
Acidobacteriota
Pseudomonadota
Actinomycetota
Planctomycetota
Dormibacterota
Campylobacterota
Desulfobacterota
DUMD01
Bacillota_G
Bacillota_C
Bacteroidota
Verrucomicrobiota
Patescibacteria
JAGRBM01
PM
Control
0.6
0.4
0.2
0
−0.2
−0.4
−0.6
Group
PM
Control
